# Improving agreement of ASA physical status class between pre-anesthesia screening and day of surgery by adding institutional-specific and ASA-approved examples: a quality improvement project

**DOI:** 10.1186/s13741-020-00162-4

**Published:** 2020-11-19

**Authors:** Amr E. Abouleish, Sandhya R. Vinta, Sarah M. Shabot, Nikul V. Patel, Erin E. Hurwitz, Partha Krishnamurthy, Michelle Simon

**Affiliations:** 1 Department of Anesthesiology, University of Texas Medical Branch, Medical Branch, 301 University Blvd. Rt 0877, Galveston, TX 77555 USA; 2 Affiliated Anesthesiologists, LLC, Oklahoma City, OK 73120 USA; 3 Bauer College of Business, University of Houston, Houston, TX 77204 USA

**Keywords:** Perioperative care, Preoperative care, Anesthesia, Physical Status Classification, Quality improvement

## Abstract

**Background:**

A successful anesthesia pre-assessment clinic needs to identify patients who need further testing, evaluation, and optimization prior to the day of surgery to avoid delays and cancelations. Although the ASA Physical Status Classification system (ASA PS) has been used widely for over 50 years, it has poor interrater agreement when only using the definitions. In 2014, ASA-approved examples for each ASA physical status class (ASA PS). In this quality improvement study, we developed and evaluated the effectiveness of institutional-specific examples on interrater reliability between anesthesia pre-anesthesia clinic (APAC) and the day of surgery evaluation (DOS).

**Methods:**

A multi-step, multi-year quality improvement project was performed. Step 1, pre-intervention, was a retrospective review to determine the percentage agreement of ASA PS assignment between APAC and DOS for adult and pediatric patients. Step 2 was a retrospective review of the step 1 cases where the ASA PS assignment differed to determine which medical conditions were valued differently and then develop institutional-specific examples for medical conditions not addressed by ASA-approved examples. Step 3 was to educate clinicians about the newly implemented examples and how they should be used as a guide. Step 4, post-intervention, was a retrospective review to determine if the examples improved agreement between APAC and DOS ASA PS assignments. Weighted Kappa coefficient was used to measure of interrater agreement excluding chance agreement.

**Results:**

Having only ASA PS definitions available, APAC and DOS agreement was only 74% for adults (*n* = 737) and 63% for pediatric patients (*n* = 216). For adults, 20 medical co-morbidity categories and, for pediatric patients, 9 medical co-morbidity categories accounted for > 90% the differences in ASA PS. After development and implementation of institutional-specific examples with ASA-approved examples, the percentage agreement increased for adult patients (*n* = 795) to 91% and for pediatric patients (*n* = 239) to 84%. Weighted Kappa coefficients increased significantly for all patients (from 0.62 to 0.85, *p* < .0001), adult patients (from 0.62 to 0.86, *p* < .0001), and pediatric patients (from 0.48 to 0.78, *p* < .0001).

**Conclusions:**

ASA-approved examples do not address all medical conditions that account for differences in the assignment of ASA PS between pre-anesthesia screening and day of anesthesia evaluation at our institution. The process of developing institutional-specific examples addressed the medical conditions that caused differences in assignment at one institution. The implementation of ASA PS examples improved consistency of assignment, and therefore communication of medical conditions of patients presenting for anesthesia care.

## Background

An effective anesthesia preoperative evaluation system’s major goal should be to medically optimize the patient and therefore reduce anesthesia risks and improve care provided. A successful system accomplishes this goal in a cost-effective manner by utilizing non-anesthesiologists, minimizing unnecessary testing and imaging, obtaining appropriate consultations, and reducing same-day cancelations (Fischer 1996). In the 20 years since Fischer first described creating an anesthesia pre-assessment clinic (APAC), the basic structure has not changed. Currently, the focus is not only on identifying and evaluating patients with medical co-morbidities, but also medically optimizing these patients in the pre-procedure period to minimize perioperative complications (Correll et al. 2006; Boudreaux and Vetter 2016; Aronson et al. 2018). APACs utilize different types of providers, where non-anesthesia clinicians (registered nurses, nurse practitioners, physician assistants, and non-anesthesiologist physicians) screen patients that are healthy to those with only mild medical conditions, while anesthesiologists become involved with the clinical decision-making for more medically complex patients. Often, this algorithm that dictates when an anesthesiologist is consulted also includes the complexity of the surgical procedure (Vetter et al. 2017; Shah and Vetter 2018).

An essential factor for the success of any APAC is that the APAC staff and the day of surgery (DOS) anesthesiologists have the same understanding of which patients need further evaluation and medical optimization. Most commonly, the severity of the patient’s medical co-morbidities is rated by using ASA Physical Status Classification system (ASA PS). Although ASA PS has been used for over 50 years and is widely accepted, utilizing only the ASA PS definitions has been shown to have poor interrater agreement (Owens et al. 1978; Haynes and Lawler 1995; Ranta et al. 1997; Mak et al. 2002; Ragheb et al. 2006; Alpin et al. 2007; Burgoyne et al. 2007; Hurwitz et al. 2017) and, therefore, can lead to the DOS anesthesiologists delaying or canceling surgeries for further evaluations and optimization. In 2014, the ASA adopted examples for each ASA PS (ASA-approved examples) to provide additional information to help guide clinicians (Abouleish et al. 2015; ASA Physical Status Classification System 2019). The use of the ASA-approved examples was effective in improving interrater agreement among anesthesia-trained and non-anesthesia-trained clinicians for hypothetical cases (Hurwitz et al. 2017).

Following the publishing of the ASA-approved examples, we conducted a multi-year, multi-step quality improvement project focused on improving agreement of the ASA PS assignment between APAC and DOS by including the ASA-approved and institutional-specific examples.

## Methods

Our Institutional Review Board approved this multi-year, multi-step retrospective quality review study and approved that no informed consent was necessary. The study was broken down into 4 periods:

- *Pre-intervention*. Identify the agreement percent of the ASA PS assignment between anesthesia pre-assessment clinic (APAC) and day of anesthesia evaluation (DOS)
- *Identify co-morbidities and develop institutional-specific examples*. Determine what co-morbidities accounted for disagreement between APAC and DOS, and if ASA-approved examples did not address, then develop additional institutional-specific examples to address these co-morbidities
- *Education*. Educate clinicians of ASA PS definitions, ASA-approved examples, and institutional-specific examples
- *Post-intervention*. Reassess the agreement percent of ASA PS assignment between APAC and DOS

### Anesthesia pre-assessment clinic (APAC)

During the study time periods, our APAC was staffed daily by one faculty anesthesiologist, one to two anesthesiology residents (CA-1), and two to three nurses (RN). The assessments were either chart reviews or chart reviews and telephone patient interviews. The faculty anesthesiologist was consulted when a patient had multiple co-morbidities, was ASA III and having moderate to high-risk surgeries, was ASA IV, or needed further evaluation and/or medical optimization. All new residents and nurses were provided with an introductory presentation and guidelines developed in APAC relating to proper assessments and indications for ordering further tests. For the pre-intervention time period, only the definitions were available to clinicians and the ASA-approved examples did not exist.

### Pre-intervention: identify the agreement percent

All elective surgical patients who had surgery from July 1 to August 31, 2013, were identified. Patients who had an assessment by APAC were included. Patients were excluded if no ASA PS was assigned in the APAC assessment. In addition, APAC did not review add-on patients (even if ambulatory), inpatients, or patients in the prison hospital; therefore, these patients were excluded.

All patients were assigned ASA PS in APAC. On the day of anesthesia, the faculty anesthesiologist made the final ASA PS as part of the pre-anesthesia evaluation (DOS).

For all patients meeting inclusion criteria, the APAC, and DOS ASA PS, and whether the patient was pediatric (less than 18 years of age) or adult (18 years and older) were recorded. If the ASA PS was different for APAC and DOS, then it was noted if APAC or DOS had a higher ASA PS. If the percentage difference was greater than 20% (Landis and Koch 1977), then the quality study continued to next period.

### Determine co-morbidities that accounted for different ASA PS assignment

For patients that had different APAC and DOS ASA PS, an investigator reviewed the patient’s medical record to determine if the difference was due to new information on DOS or if there was no new information, which medical co-morbidities were judged differently. The reviewer could identify more than one co-morbidity if applicable. If the initial reviewer could not find clear reasons for the difference, then two additional investigators reviewed the medical record and the three investigators determine why the ASA PS was assigned differently or if there was no reason.

Each reviewer was asked to describe, in free text, the patient’s co-morbidities and his/her evaluation of the difference of the valuation of the ASA PS by APAC and DOS. The free texts were then reviewed and grouped into categories of medical conditions. For example, for one patient, mild COPD was valued differently, and in another, severe COPD on home oxygen was valued differently. The two patients were combined into one co-morbidity category, COPD.

In each group (pediatric and adult), co-morbidity categories that accounted for > 1% of the patients with different ASA PS were identified. If the ASA-approved examples do not address the co-morbidity, then institutional-specific examples were developed to address the range of severity a co-morbidity can present with and the appropriate ASA PS. For instance, in the example of COPD, “mild lung disease” is an ASA-approved example for ASA II and “COPD” is an ASA-approved example for ASA III. In this example, institutional-specific examples might be developed to define “home oxygen therapy” as ASA III.

A draft table with ASA-approved and institutional-specific examples was presented to the clinical faculty of the Department of Anesthesiology in our institution for feedback. Adjustments were made to create a final table.

### Educate clinicians

In the APAC, the final table was included in the orientation packet for the resident’s rotation. It was posted at all anesthesiology resident and APAC nurse work stations within the clinic. The final table was presented in departmental conferences, included in didactic presentations (to staff about pre-anesthesia assessment), and was emailed to all clinical faculty, residents, and nurse anesthetists.

### Post-intervention: reassess agreement percent between APAC and DOS

Similar to the first phase methods, all elective surgical patients who had surgery from July 1 to August 31, 2016, were identified. These 2 months correspond to the same time period as was evaluated in the pre-intervention period and took place 8 months after education was initiated. Inclusion and exclusion criteria used in the pre-intervention period were also used in the post-intervention period.

All patients were assigned ASA PS in APAC. On the day of anesthesia, the faculty anesthesiologist made the final ASA PS as part of the pre-anesthesia evaluation (DOS).

For all patients meeting inclusion criteria, the APAC ASA PS and DOS ASA PS, and whether the patient was pediatric (less than 18 years of age) or adult (18 years and older) were recorded.

### Statistical analysis

In both pre-intervention period and post-intervention period, the percent agreement was calculated by dividing the number of patients with different ASA PS for APAC and DOS by the total number of patients in all patients, adult patients, and pediatric patients.

Cohen’s weighted Kappa coefficient were determined for all cases and for each group (adult, pediatric). Kappa coefficient is statistical test to describe strength of interrater agreement. The weighted Kappa coefficient describes agreement excluding chance agreement by weighing the degree of disagreement (Landis and Koch 1977; McHugh 2012). The strength of agreement is categorized by the Kappa coefficient in the following manner: Kappa value 0–0.20 = slight agreement, 0.21–0.39 = fair agreement, 0.40–0.59 = moderate agreement, 0.60–0.79 = good agreement, and 0.80–1.0 = very good agreement (Altman 1991). For adequate agreement, the Kappa value should be 0.60 or higher. Kappa coefficient is with 95% confidence intervals (mean ± 1.96*ASE). Chi square tests on Kappa coefficient were used to determine if there was a statistical difference in interrater agreement between the pre-intervention and post-intervention groups (Fleiss et al. 2004).

## Results

### Pre-intervention

**Fig. 1:**
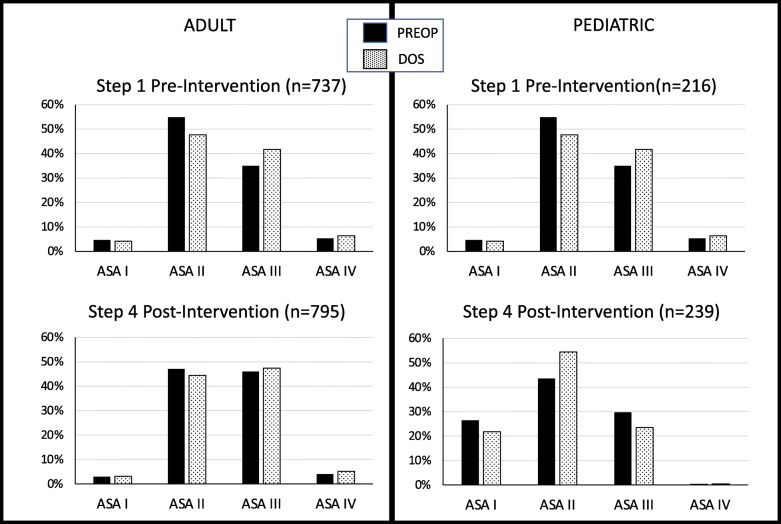
Distribution of ASA PS assignment. In the pre-intervention period, only the ASA PS definitions were available. In the post-intervention period, in addition to the definitions, ASA-approved and institutional-specific examples were available. APAC ASA PS assignment was done based on chart review with or without telephone interview by anesthesiology faculty, anesthesiology resident, or non-anesthesia-trained nurse working in pre-anesthesia assessment clinic. DOS ASA PS assignment made by the anesthesiologist as part of the pre-anesthesia evaluation on the day of the surgery

In the pre-intervention period, 953 patients (737 adult and 216 pediatric patients) met inclusion criteria. For both groups, patients were classified ASA I to IV with no ASA V. Pediatric patients had higher percentage of ASA I while the adult patients had a higher percentage of ASA III (Fig. 1).

**Fig. 2:**
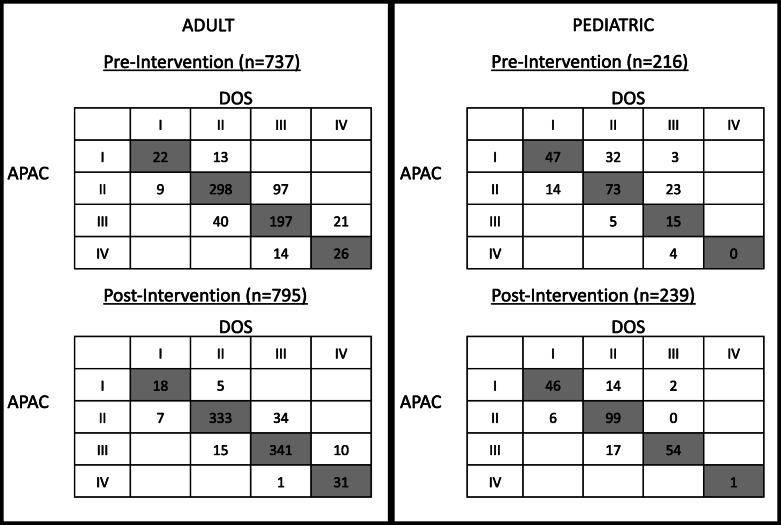
ASA PS assignment APAC vs. DOS. In the post-intervention period, with the addition of ASA-approved and institutional-specific examples, there is more agreement between APAC and DOS than in the pre-intervention period. When there was a different ASA PS assignment, in almost all cases it was within 1 level

ASA PS assignment between APAC and DOS was the same for 74% of adult patients and 63% of pediatric patients. In both adults and pediatrics, when the ASA PS was different, more than two thirds had a higher ASA PS on DOS than APAC (Table 1). Further, in almost all patients with different ASA PS assignment, the APAC and DOS only differed by 1 (Fig. 2).

**Table 1:** ASA PS APAC vs. DOS

	*n*	APAC = DOS, *n* (%)	APAC > DOS, *n* (%)	APAC < DOS, *n* (%)
Pre-intervention				
Adult	737	543 (74%)	63 (9%)	131 (18%)
Pediatric	216	135 (63%)	23 (11%)	58 (27%)
Post-intervention				
Adult	795	723 (91%)	23 (3%)	49 (6%)
Pediatric	239	200 (84%)	23 (10%)	16 (7%)

In the pre-intervention time period, only the ASA PS were available at that time. In the post-intervention time period, in addition to the definitions, clinicians were educated to ASA-approved and institutional-approved examples

*ASA PS* ASA physical status class, *APAC* anesthesia pre- assessment clinic, *DOS* day of surgery anesthesiologist

**Fig. 3:**
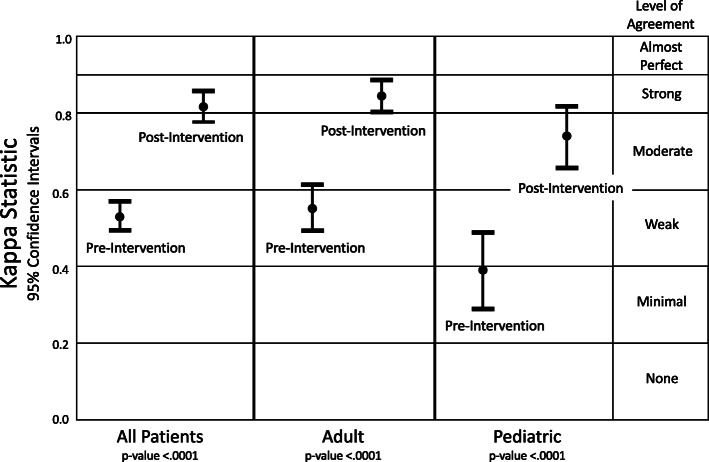
Interrater agreement measured with weighted Kappa coefficient and 95% confidence intervals. The level of agreement for post-intervention was moderate to strong and significantly higher than pre-intervention for all patients and for adult and pediatric groups. Weighted Kappa measures interrater agreement excluding chance agreement

Weighted Kappa coefficient for all patients, adult, and pediatric groups were 0.62 (0.58–0.66), 0.62 (0.57–0.67), and 0.47 (0.38–0.57), respectively (Fig. 3).

### Institutional-specific examples and education

Because the difference in ASA PS met the threshold, we continued on with the quality improvement project. From pre-intervention period, 274 patients with 194 adult and 81 pediatric were identified as having different ASA PS assigned by APAC and DOS. In 10% of adult and 11% of pediatric patients, the information available for DOS was different than that provided when APAC assigned the ASA PS. Therefore, 173 adult and 72 pediatric patients’ APAC and DOS evaluations were reviewed to identify which medical co-morbidities were valued differently by APAC and DOS.

In 89% of the 173 patients, the reason DOS assigned a different value for ASA PS than APAC was identified during the initial review. But in 27 patients (11%), additional investigators reviewed the records and developed consensus as to why the ASA PS was valued differently. The percentage of patients needing further review was similar between adult (12%) and pediatric (10%).

Although patients may have more than one underlying medical co-morbidity, the investigators focused on identifying which co-morbidities were responsible for differing ASA PS assignment. In 64 patients (26%), more than one co-morbidity was identified as being valued differently by APAC and DOS. Adult patients had a higher percentage (28%) of instances with more than one co-morbidity than pediatric patients (21%). Because of these multiple co-morbidities, the total co-morbidities identified are greater than the number of patients: 235 co-morbidities in adult and 95 in pediatric patients.

For adult patients, of all the co-morbidities identified as the reason the ASA PS was valued differently, 19 co-morbidities occurred in more than 1% of co-morbidities (> 2 patients) (Table 2). Of these 19 co-morbidities, body mass index (BMI), hypertension, obstructive sleep apnea (OSA), cancer (history of or active), peripheral vascular disease, and the use of tobacco products each occurred more than 5% of the time. In 4% (8 patients), the only reason the ASA was valued differently was due to the advanced age of the patient. In 4% (8 patients), the reason the ASA PS was valued differently appeared to be due to multiple mild systemic diseases. Only 9 of the 19 categories are identified in ASA-approved examples, the other 10 are not included.

**Table 2:** Adult and Pediatric medical co-morbidities associated with different ASA PS assignment, step 2

	Adult (*n* = 235)	ASA-example		Pediatric (*n* = 95)	ASA-example
BMI	16%	Y	URI (includes sinusitis, adenoiditis) or OM	21%	N
HTN	13%	Y	OSA	21%	N
OSA	6%	N	Developmental delay (motor or cognitive)	16%	N
Cancer	6%	N	PCA in premature infant	8%	Y
PVD	6%	N	Asthma	6%	N
Tobacco	5%	Y	Dental caries	6%	N
Advanced age	4%	N/A	Full term valued as ASA 2	4%	N
CAD	4%	Y	Psychiatric conditions (bipolar, anxiety d/o, depression, autism)	3%	N
COPD	4%	N	Allergic rhinitis	6%	N
Dysrhythmia (atrial fibrillation, SVT pacemaker, AICD)	4%	Y	Anemia	1%	N
ESRD	4%	Y	Congenital heart disease	1%	N
Multiple co-morbidities	4%	N/A	Imperforate anus	1%	N
DM	3%	Y	Laryngeal papilloma	1%	N
CKD	3%	N	Craniosynostosis	1%	N
Psychiatric (bipolar, anxiety disorder, depression, autism)	2%	N	Scoliosis	1%	N
Anemia	2%	N			
GERD	2%	N			
Pulmonary HTN	2%	N			
CVA/TIA	2%	Y			
Allergic rhinitis	.5%	N			
Asthma	.5%	Y			
Down	.5%	N			
Recent URI	.5%	N			

In Step 2, medical co-morbidities were identified as being valued differently by PREOP and DOS. In 28% of adult patients, more than one co-morbidity was identified; hence, the total number of co-morbidities is higher than number of patients. In 21% of pediatric patients, more than one co-morbidity was identified; hence, the total number of co-morbidities is higher than number of patients. For medical co-morbidity that occurred more than 1%, institutional-specific examples were developed if the ASA-approved examples did not address it. No example was developed for advanced age or multiple medical co-morbidities since the department anesthesiologists did not have consensus on how these should be handled. Unlike adult patients’ co-morbidities, almost all pediatric co-morbidities are not included in the ASA-approved examples

*AICD* automatic implantable cardiac defibrillator, *ASA PS* ASA physical status class, *BMI* body mass index, *CAD* coronary artery disease, *CKD* chronic kidney disease, *COPD* chronic obstructive pulmonary disease, *CVA* cerebrovascular accident, *DM* diabetes mellitus, *ESRD* end-stage renal disease, *GERD* gastroesophageal reflux disease, *HTN* hypertension, *OSA* obstructive sleep apnea, *PCA* post-conceptual age, *PVD* peripheral vascular disease, *SVT* supraventricular tachycardia, *TIA* transient ischemic attack, *URI* upper respiratory infection

For pediatric patients, of all the co-morbidities identified as the reason the ASA PS being valued differently, 9 co-morbidity categories occurred in more than 1% of co-morbidities (> 1 patient) (Table 2). The co-morbidity of upper respiratory infection (URI) accounted for 21% of the co-morbidities and included the sub-categories of ongoing or recent URI (3%), chronic otitis media (15%), and chronic sinusitis or adenoiditis (3%). OSA in pediatric patient presenting for tonsillectomy also accounted for 21%. Developmental delays (motor or cognitive) accounted for 16% of the co-morbidities and included a spectrum of conditions including cerebral palsy (6%), Down syndrome (5%), and tracheostomy-dependent patients who are presenting from home (rather than inpatient facility) (5%). Similar to adult patients, in 4% (3 patients), the reason the ASA PS was valued differently was age < 1 year in former full-term infants. Except for former premature infants post-conceptual age < 60 weeks, none of these co-morbidities are addressed in the ASA-approved examples.

Using the co-morbidities identified, institutional-specific examples for the co-morbidities and ASA PS assignment were developed. These examples were distributed to clinical faculty and discussed at the departmental morbidity and mortality conference. After feedback to the examples, a final set of examples was developed as shown in Table 3.

**Table 3:** ASA-approved and institutional-specific examples for ASA PS

ASA PS	Definition	Descriptor (by ASA)	Cardiac	Pulmonary	Renal	Endocrine/GI	Other	Pedi	OB
**ASA I**	A normal healthy patient						*All ages*	*All ages except premature infants*	
**ASA II**	A patient with mild systemic disease	Mild diseases only without substantive functional limitations	Well controlled HTN; *mild pulmonary HTN*	Current smoker; mild lung disease *(asthma, COPD, or OSA)*	*Chronic kidney disease (GFR > 44 ml/min)*	Obesity (30 < BMI < 40); well controlled DM; *mild GERD*	Social alcohol drinker; *History (> 3 months) of cancer unrelated to surgery; psychiatric does not affect daily living*	*URI > 2 weeks; mild asthma; allergic rhinitis; dental caries; sleep-disturbed breathing (snoring); mild developmental delay (motor or mental)*	Uncomplicated pregnancy
**ASA III**	A patient with severe systemic disease	Substantive functional limitations; one or more moderate to severe diseases	Poorly controlled HTN; implanted pacemaker; moderate reduction in ejection fraction; history (> 3 months) of MI, or CAD/stents; *moderate to severe pulmonary HTN*	*Moderate to severe asthma, COPD, or OSA; on CPAP; on home oxygen*	*Chronic kidney disease (GFR < 44 ml/min);* ESRD undergoing regularly scheduled dialysis	Morbid obesity (BMI ≥ 40); active hepatitis; alcohol dependence; *moderate to severe GERD*	History (> 3 months) of TIA or CVA *cancer related to surgery; recent (< 3 months) cancer unrelated to surgery; psychiatric affects daily living*	Former premature infant with PCA < 60 weeks; *URI < 2 weeks; Moderate to severe asthma, OSA; moderate to severe developmental delay (motor or mental)*	*Complicated pregnancy*
**ASA IV**	A patient with severe systemic disease that is a constant threat to life		Recent (< 3 months) MI, or CAD/stents; ongoing cardiac ischemia or severe valve dysfunction; severe reduction of ejection fraction	ARD	ESRD not undergoing regularly scheduled dialysis		Recent (< 3 months) CVA or TIA; sepsis; DIC		

Institutional-specific examples were determined in step 2 in reviewing patients with different ASA PS class between PREOP and DOS. The examples were presented and commented on by departmental anesthesiologists. The final institutional-specific examples are italized in the table. ASA-approved examples are not underlined. In step 3, this table was used in education of clinicians (anesthesiologists, residents, nurse anesthetists, nurses in the pre-anesthesia clinic) and posted in the pre-anesthesia clinic

*ASA PS* ASA physical status class, *BMI* body mass index, *CAD* coronary artery disease, *COPD* chronic obstructive pulmonary disease, *CPAP* continuous positive airway pressure, *CVA* cerebrovascular accident, *DIC* disseminated intravascular coagulation, *DM* diabetes mellitus, *ESRD* end-stage renal disease, *GERD* gastroesophageal reflux disease, *GFR* glomerular filtration rate, *HTN* hypertension, *MI* myocardial infarction, *OSA* obstructive sleep apnea, *PCA* post-conceptual age, *TIA* transient ischemic attack, *URI* upper respiratory infection

### Post-intervention

For the post-intervention period, 1034 patients (795 adult and 239 pediatric patients) met inclusion criteria. For both groups, patients were classified ASA I to IV with no ASA V (Fig. 2). The distribution of DOS assigned ASA PS for post-intervention was similar to pre-intervention (Fig. 1).

The percentage of same ASA PS assignment between APAC and DOS increased in the post-intervention period (Table 1). The percentage of adult patients with the same APAC and DOS increased from 74 to 91%. The percentage of pediatric patients with same APAC and DOS increased from 63 to 84%.

Weighted Kappa coefficients were in the “Very Good Agreement” range for all patients (0.85 (0.82–0.87)) and adult patients (0.86 (0.83–0.89)), and in the “Good Agreement” range for pediatric patients (0.78 (0.72–0.84)) (Fig. 3). Compared to the pre-intervention group, the Weighted Kappa coefficients increased significantly in the post-intervention group for all patients (*p* < .0001), adult patients (*p* < .0001), and pediatric patients (*p* = .0003).

## Discussion

Although it has inherent deficiencies, the ASA PS system continues to be used due to its historical use and simplicity. The purpose of the ASA PS system is to communicate pre-existing medical conditions (Mayhew et al. 2019). Improving understanding and developing a consensus is important for each anesthesiology department to have meaningful communication within the anesthesiology group and to non-anesthesia-trained clinicians at the same institution.

Pre-anesthesia assessment processes incorporate initial screening and chart reviews by non-anesthesia-trained clinicians. The ASA PS assignment is used as guidelines to determine which patient is to be assessed in the pre-anesthesia period by an anesthesiologist, as well as whether the patient should be seen in person versus having only a telephone encounter or chart review (Fischer 1996; Correll et al. 2006; Boudreaux and Vetter 2016; Aronson et al. 2018; Vetter et al. 2017; Shah and Vetter 2018). Vetter et al. describe one possible process that entitled “PASS-GO” (Vetter et al. 2017; Shah and Vetter 2018). In PASS-GO, the ASA PS is matched with the surgical procedure risk-intensity. For all ASA I and II patients with low-risk procedure planned, only a telephone interview by a nurse is done (EXPRESS). Preoperative assessment (PASS) is done on ASA II patients with high-risk procedure planned and ASA III and IV patients when the low-risk procedure is planned. This assessment includes face-to-face assessment in the clinic by a non-physician provider and may also include testing, but consultations are not normally needed. In the ASA III and IV patients having high-risk procedures, global optimization (GO) is performed which involves face-to-face evaluation by anesthesiologist and possibly multiple consultations are considered as well as further investigational testing. Again, if the ASA PS is not correctly assigned in APAC, then the patient may not receive the proper pre-anesthesia assessment and optimization.

ASA PS is a guideline for determining which patients need further testing, as well as need for additional evaluation by anesthesiologist and other specialties. In the Choose Wisely initiative, the first recommendation is “Don’t obtain baseline laboratory studies in patients without significant systemic disease (ASA I or II) undergoing low-risk surgery – specifically complete blood count, basic or comprehensive metabolic panel, coagulation studies when blood loss (or fluid shifts) is/are expected to be minimal” (Choosing Wisely: American Society of Anesthesiologists 2020).

Until 2014, only the ASA PS definitions were available for a clinician to make the assignment. Although in hypothetical cases, the ASA PS assignments by respondents agreed with the investigators’ “correct” assignment less than 70% of the time for some and even less than 20% in some cases (Owens et al. 1978; Haynes and Lawler 1995), no change in the definitions were made mainly for the consensus that anesthesiology training and experience should be relied on for making the final assignment after evaluating the patient in person.

Many pre-anesthesia assessment clinics are being staffed by non-physician clinicians who are not anesthesia-trained but are still making initial ASA PS assignment; Sankar et al. showed that only 67% of assignments made in the pre-anesthesia clinic was the same as that made on the day of surgery by the anesthesiologist (Sankar et al. 2014). ASA PS is also being used by non-anesthesia clinicians as guidelines for when it might be appropriate for non-anesthesia clinicians to provide moderate sedation and in which facility to have a particular procedure (American Society for Gastrointestinal Endoscopy 2008; Jarzyna et al. 2011; Oldham 2014; Hinkelbein et al. 2018; Zielinska et al. 2019). Since these individuals performing the assessments are not anesthesia-trained, one cannot rely on training to supplement the existing definitions to help guide proper ASA PS assignments.

We also found that when using ASA PS definitions, more than 20% of the patients did not have the same ASA PS assignment in both APAC and DOS. For example, in the pre-intervention period for adult patients, 19% of the patients were assigned ASA II in APAC and assigned ASA III on DOS (Fig. 2). In other words, if the APAC clinician would not have consulted an anesthesiologist for further evaluation and if the clinician followed the Choose Wisely guidelines, no additional laboratory tests would be ordered. This may have led to a situation in which the DOS anesthesiologist wanted additional tests prior to proceeding and may even result in delaying the procedure for the test results or canceling the procedure if the tests are abnormal.

For these reasons, the ASA-approved examples to each of the ASA PS to be a guide to all clinicians whether they might be anesthesia and non-anesthesia-trained. Hurwitz et al. examined the impact of including the ASA-approved examples in addition to the ASA PS definitions on ASA PS assignment by both anesthesia- and non-anesthesia-trained clinicians for hypothetical cases (Mak et al. 2002). Similar to previous studies, using only definitions, the overall percentage of correct assignments (as defined by the investigators) was less than 60% with some cases having less than 30% correct assignments. Using the definitions, anesthesia-trained clinicians had significantly higher correct assignments than the non-anesthesia clinicians. When the ASA-approved examples were included, the correct assignment rate increased to nearly 80% in both anesthesia-trained and non-anesthesia-trained clinicians and no difference between groups. Further, they found those cases with lower percentage correct assignments did not have a medical co-morbidity listed in the ASA-approved examples.

In this multi-year, multi-step quality improvement project, in the pre-intervention period, we performed a study similar to Haynes et al.’s study to determine if we too had low interrater agreement between APAC and DOS (Haynes and Lawler 1995). Because the APAC assessment is used by the DOS anesthesiologist, we hoped that the agreement between APAC and DOS would be high and no intervention would be needed. But because we also had similar rates of less than 70%, we proceeded to try to improve the assignments by utilizing examples for each ASA PS. In reviewing the ASA-approved examples, we recognized that these examples might not address all the common medical co-morbidities that *our patients* had; hence, the second phase was designed to identify additional institutional-specific examples to supplement the ASA-approved examples. After education phase, we found in the post-intervention period there was improvement in interrater agreement of the ASA PS assignment between APAC and DOS.

Almost all the ASA-approved examples describe co-morbidities normally seen in the adult patient population and do not address many of the co-morbidities (both congenital defects and diseases) found in the pediatric population. Although most pediatric patients presenting for surgery at our institution do not have moderate to severe chronic medical conditions (ASA III or higher), there are common medical co-morbidities seen in our patients. In our clinical practice, the APAC assignment was done by a non-pediatric anesthesiology trained clinician while most of the DOS evaluations are completed by a pediatric anesthesiologist. By only using the ASA PS definitions, almost 40% of the patients had different ASA PS assignment on DOS as compared to the APAC.

Poor interrater agreement for pediatric anesthesia hypothetical cases has been demonstrated (Ragheb et al. 2006; Alpin et al. 2007; Burgoyne et al. 2007). Recently, anesthesiologists from Boston Children’s Hospital describe a similar process as our pre-intervention period and developing institutional-specific examples to help develop consistency among their clinicians (Leahy et al. 2019). Many of the examples developed were not included in our institutional-specific examples. This difference highlights different populations. For example, Boston Children’s Hospital is a leading center for congenital heart care (surgical and medical), while at our institution we do not provide primary congenital heart care. Therefore, it is not surprising that their examples included congenital heart co-morbidities while ours did not. This difference highlights the importance of supplementing the ASA-approved examples with institutional-specific examples. We would expect that a cancer hospital would have more cancer-related institutional-specific examples, and an adult heart center would have more cardiovascular-related examples. Although these institutional-specific examples may not be universally approved by the ASA or all anesthesiologists, they represent a consensus within an anesthesiology group.

By developing institutional-specific examples to supplement the ASA-approved examples, the surgeons and other proceduralists can use this tool to better understand when a patient would be ASA III or higher and may need further testing and optimization prior to the procedure. In addition, when a patient is ASA I and II, they would better understand that this patient would not likely require further work-up. It will help set expectations for both the surgeon and the patient about the pre-anesthesia assessment process.

Even going through a quality improvement project, sometimes consensus among anesthesiologists in the same department or facility are not possible for all conditions. For example, a patient with multiple mild systemic diseases might be considered ASA III by some anesthesiologists and ASA II by the definitions and by other anesthesiologists. In our study, this was one of the reasons for differences found when we reviewed pre-intervention cases. After robust debate, we decided to not include the number of mild systemic diseases as a criterion for ASA III.

There are several limitations to our quality improvement project. First, by the nature of quality improvement study, we did not undertake either a sample size analysis in the pre-intervention period, but instead arbitrarily chose the time period of 2 months to review to see if there was a problem that needed to be improved. Despite this lack of sample size, we found low agreement percentages similar to published studies. This 2-month period also limited our evaluation of post-intervention data collection to the same 2-months of the calendar year. Second, our institutional-specific examples identified in this study and the ASA-approved examples are not all the medical co-morbidities seen in our patients. The quality improvement project focused on agreement of ASA PS assignment between APAC and DOS. Since we did not examine the medical records of patients who had the same ASA PS for APAC and DOS, we cannot comment on medical co-morbidities in those patients. In other words, there may be more co-morbidities that are not included in the examples but are not needed since there appears to be agreement on the co-morbidities impact on ASA PS. Third, similarly, inpatients were excluded from the study since they did not have an APAC ASA PS assignment. Medical co-morbidities associated uniquely to inpatients, e.g., necrotizing enterocolitis or acute respiratory distress syndrome, would not be identified in our study. Fourthly, because the institutional-specific examples were for medical co-morbidities where clinicians valued the impact on ASA PS differently, by definition, these examples and the final assignment may be debatable. We felt that consensus over the institutional-specific examples allowed for consistent communication at our institution.

Finally, the definitions, ASA-approved examples and the institutional-specific examples are only guidelines. The final assignment has to be done by the anesthesiologist during his/her pre-anesthesia evaluation. Although this fact may cause dismay in purists who would prefer universally accepted assignment of ASA class on every patient, the reality is that patients are not always that black and white. In other words, although medicine is a science, it also an art.

## Conclusions

In summary, this multi-step, multi-year quality improvement project demonstrates that definitions and ASA-approved examples of ASA PS benefit with institutional-specific examples for ASA PS. In developing these examples, important debate and consensus-building on common co-morbidities seen by that department of anesthesiology leads to more consistent institutional understanding of ASA PS assignment. The consistent assignment of ASA PS is important to communicate among anesthesiologists, other anesthesia clinicians, surgeons, and other clinicians involved in the perioperative care.

## Data Availability

Please contact author for data requests.

## Abbreviations

ASA: American Society of Anesthesiologists
ASA PS: ASA Physical Status Classification
APAC: Anesthesia Pre-assessment Clinic
DOS: Day of Surgery (or Anesthesia) Anesthesiologist
BMI: Body mass index
CAD: Coronary artery disease
CKD: Chronic kidney disease
COPD: Chronic obstructive pulmonary disease
CPAP: Continuous positive airway pressure
CVA: Cerebrovascular accident
DIC: Disseminated intravascular coagulation
DM: Diabetes mellitus
ESRD: End-stage renal disease
GERD: Gastroesophageal reflux disease
GFR: Glomerular filtration rate
HTN: Hypertension
MI: Myocardial infarction
OSA: Obstructive sleep apnea
PCA: Post-conceptual age
PVD: Peripheral vascular disease
SVT: Supraventricular tachycardia
TIA: Transient ischemic attack
URI: Upper respiratory infection
